# The Long-term Impact of Childhood Experiences on Cognitive Function in Later Life: Evidence From the HCAP dataset

**DOI:** 10.1093/geroni/igaf122.1585

**Published:** 2025-12-31

**Authors:** Yujun Liu, Thomas Smith, Jamie Mayer

**Affiliations:** Northern Illinois University, Naperville, Illinois, United States; Northern Illinois University, DeKalb, Illinois, United States; Northern Illinois University, DeKalb, Illinois, United States

## Abstract

**Objectives:**

This study aims to explore the long-term impact of childhood experiences on cognitive function in later life and examines the moderating role of physical activity and educational attainment in adulthood.

**Methods:**

Using the first wave of the Harmonized Cognitive Assessment Protocol (HCAP) data, our sample included adults aged 65 or older in the U.S. (N = 3,496). We used multiple regression to model the relationships involving childhood experiences (parental education, childhood financial situation, and childhood health), physical activity and educational attainment in adulthood, and cognitive function in later life, specifically, memory (MEM), executive function (EF), visual-spatial cognition (VS), language and fluency (LF), and global cognition (GC).

**Results:**

The findings indicated that higher levels of financial situation in childhood and parental education positively predicted EF, LF, and GC. Participants with higher level of childhood health had higher levels of EF, GC, and VS. Participants with higher levels of educational attainment and physical activity in adulthood showed higher levels of all five cognitive domains. There was a significant positive buffering effect of physical activity on the relationship between parental education and LF. Educational attainment also significantly and positively moderated the relationship between parental education and both EF and LF. We also found a gender difference in the relationship between childhood health and LF and VS.

**Discussion:**

This study has important implications for developing recommendations and interventions that target behavioral and social factors associated with cognitive change among older adults with the greater goal of delaying or preventing Alzheimer’s diseases and related dementia.

